# TAF1A and ZBTB41 serve as novel key genes in cervical cancer identified by integrated approaches

**DOI:** 10.1038/s41417-020-00278-1

**Published:** 2020-12-12

**Authors:** Mingyuan Wang, Jingnan Liao, Jinjin Wang, Mingming Qi, Kangkai Wang, Wei Wu

**Affiliations:** 1 Department of gynaecology and obstetrics, the Affiliated Zhuzhou Hospital Xiangya Medical College, Central South University, Zhuzhou, Hunan China; 2 Department of Pathophysiology, School of Basic Medical Science, Central South University, Changsha, Hunan China; 3 Institute of Reproductive and Stem Cell Engineering, School of Basic Medical Science, Central South University, Changsha, Hunan China; 4 Key Laboratory of Sepsis Translational Medicine of Hunan, Central South University, Changsha, Hunan China; 5 Department of Laboratory Animals, Hunan Key Laboratory of Animal Models for Human Diseases, Xiangya School of Medicine, Central South University, Changsha, Hunan China; 6 Department of Geratic Surgery, Xiangya Hospital, Central South University, Changsha, Hunan China; 7 National Clinical Research Center for Geriatric Disorders, Xiangya Hospital, Central South University, Changsha, Hunan China

**Keywords:** Gynaecological cancer, RNAi

## Abstract

Cervical cancer (CC) is the second most common cancer and the leading cause of cancer mortality in women. Numerous studies have found that the development of CC was associated with multiple genes. However, the mechanisms on gene level are enigmatic, hindering the understanding of its functional roles. This study sought to identify prognostic biomarkers of CC, and explore their biological functions. Here we conducted an integrated analysis to screen potential vital genes. Candidate genes were further tested by experiments in clinical specimens and cancer cell line. Then, molecular modeling was used to predict the three-dimensional structure of candidate genes’ proteins, and the interaction pattern was analyzed by docking simulation technique. Among the potential genes identified, we found that TAF1A and ZBTB41 were highly correlated. Furthermore, there was a definite interaction between the proteins of TAF1A and ZBTB41, which was affected by the activity of the p53 signaling pathway. In conclusion, our findings identified TAF1A and ZBTB41 could serve as biomarkers of CC. We confirmed their biological function and deciphered their interaction for the first time, which may be helpful for developing further researches.

## Introduction

Cervical cancer (CC) is the second most common type of cancer affecting women [1]. It is asymptomatic in its early stages and presents various symptoms in its advanced stages, including irregular vaginal bleeding [2]. The specific cause of CC is unknown; however, the Human papillomavirus (HPV) infection, sexually transmitted infections (STIs), weakened immunity, among other risk factors, have been implicated. Some studies have suggested that CC is a complex multifactorial disease, associated with environment and genetic factors [3].

The current advances in screening and treatment methods have improved the prevention and management of CC; however, most patients are diagnosed late, thus complicating their management. The main treatment methods for CC are radical surgery and local radiotherapy, supplemented by chemotherapy, which often cause various complications, and severely affect the patients’ quality of life [4]. Therefore, it is imperative to find a sensitive and reliable method to improve diagnosis, reduce mortality rate, and explore the pathogenesis of CC.

Research has implicated the HPV infection as an independent risk factor for CC [5]. The HPV infection induces various genetic changes in the cervical tissue, which directly affect the proliferation of cancer cells, malignancy of tumours, and the prognosis of patients. In our previous study, we suggested that genetic variations may contribute to CC’s pathogenesis and identified several hub genes [6]. Similarly, the Cancer Genome Atlas Research Network identified significant alterations in the SHKBP1, ERBB3, CASP8, HLA-A, and TGFBR2 genes, in CC patients [7]. However, the contribution of the key genes and their functions in CC are unclear. As a result, further studies exploring the role of hub genes in the pathogenesis of CC and its progression are warranted.

The development of large-scale genomic technologies has promoted studies involving tumor biomarkers. The Weighted Gene Co-Expression Network Analysis (WGCNA) is among the most powerful genomic techniques used in research [8]. It applies systems biology to determine the correlation of gene expression and construct the gene modules with biological significance using a data mining algorithm. In recent years, the WGCNA technique has been extensively utilised in various diseases’ research, especially tumors, to explore vital genes and therapeutic targets [9–11].

Likewise, a more detailed in silico analysis with ab initio modeling serves as an essential and effective auxiliary method for simulating the macromolecular structure of proteins with unconfirmed crystal structures. This method has been applied in most proteins to generate new structures from sequences, crucial for protein model design and protein folding. Additionally, this technique has been widely used to analyse protein structures and intermolecular interaction patterns of various proteins [12, 13].

This study focused on the association between gene sets and the common phenotypes of CC. We identified two candidate genes (TAF1A and ZBTB41) by combining a set of complex molecular analyses. Subsequent experiments showed that the TAF1A and ZBTB41 gene expression in the tumour and normal tissues were significantly different. Moreover, the results revealed that these genes were involved in the proliferation and migration of tumour cells. Co-immunoprecipitation results suggested that TAF1A and ZBTB41 form a complex, and their binding ability was affected by the activity of the p53 signaling pathway. Subsequently, we predicted the protein structures of TAF1A and ZBTB41. Molecular docking results showed that TAF1A and ZBTB41 interacted through hydrophobic, van der Waals, hydrogen, and electrostatic bonds. This finding may have significant prognostic value for CC and unveil the potential mechanism of the interaction between TAF1A and ZBTB41 for the first time.

## Results

### The WGCNA and identification of key module

**Fig. 1:**
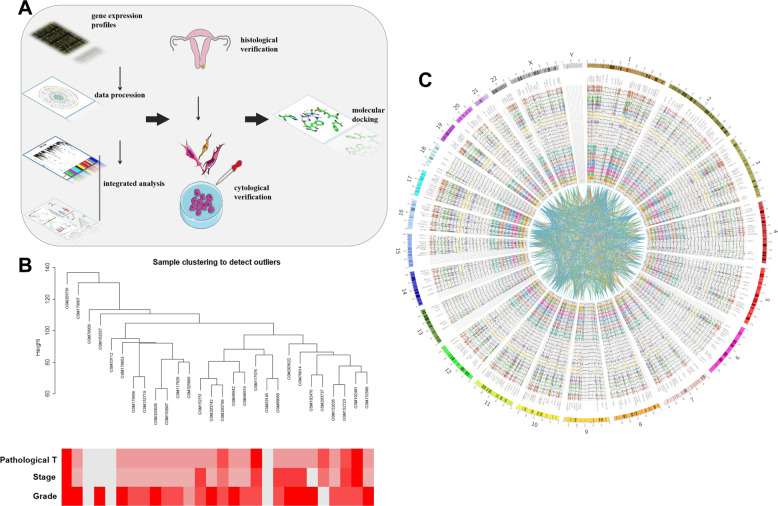
Research schematic and WGCNA data preprocessing. **A** The schematic representation of the study. **B** Clustering dendrogram and phenotypic information heatmap of 28 samples. **C** A Circos plot of the top 25% most variant mRNA expression and their co-expression network.

**Fig. 2:**
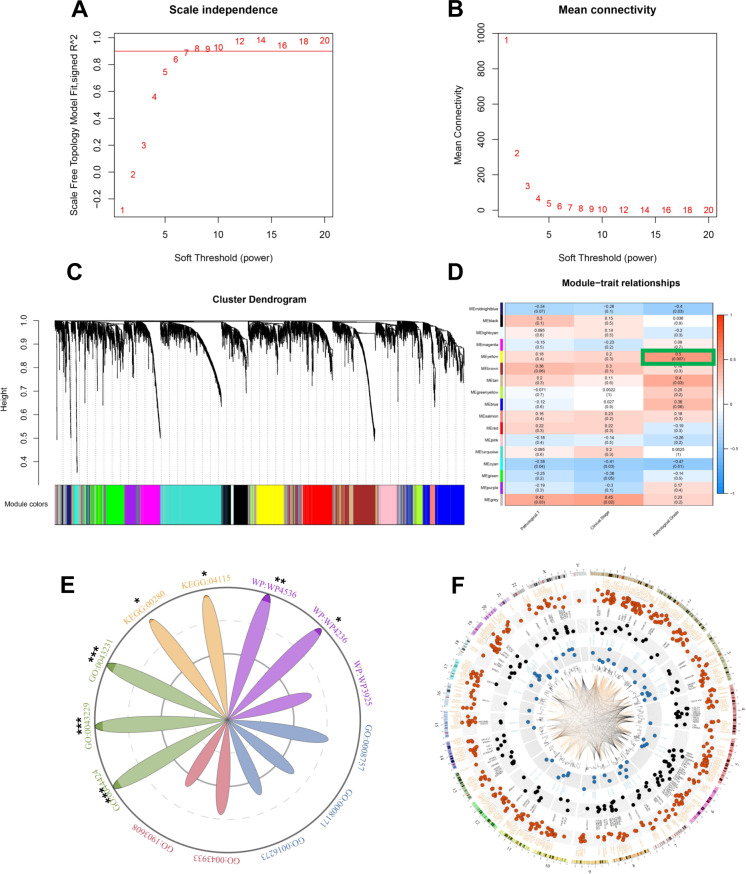
Determination of soft-thresholding power and identification of modules associated with clinical traits of CC. **A** Analysis of the scale-free fit index for various soft-thresholding powers (β). **B** Analysis of the mean connectivity for various soft-thresholding powers (β). We choose the lowest β that resulted in approximate scale-free topology. **C** Dendrogram of genes clustered based on a dissimilarity measure (1-TOM). The colour band provides a simple visual comparison of module assignments. The colour band shows the results from the automatic single block analysis. **D** Heatmap of the correlation between module eigengenes and clinical traits of CC. **E** A Radar chart of gene ontology and pathway enrichment. The Radii of each enrichment were represented by 1-P. The radius of the shadow circle is 0.95 times of the largest circle. Different categories are distinguished by colours: molecular function (blue), cellular component (red), biological process (green), Kyoto Encyclopedia of Genes and Genomes pathway (yellow), and WikiPathways (violet). *P < 0.05; **P < 0.01; ***P < 0.001. **F** The circos plot of the genes within the three most significant modules (yellow, tan and cyan).

The samples were clustered using the average linkage method, analysed using Pearson’s correlation, and the corresponding heatmap showed phenotypic information about each sample (Fig. 1B). The 25% topmost variant genes were selected for the WGCNA. The mRNA expression and co-expression were presented in a circos plot (Fig. 1C). The power of β = 8 (R^2^ = 0.918) was determined as the soft threshold to obtain a scale-free network (Fig. 2A, B). Afterward, 17 gene modules were established, and a dendrogram was used to represent the different gene modules (Fig. 2C). The co-expression relationships of the genes within the most significant three modules were presented in a circos plot (Fig. 2F). The yellow module (*P* < 0.01, Fig. 2D) was selected as a clinically significant module for further analysis. The total number of genes of the yellow module was shown in supplementary Table 1.

### Functional enrichment analysis

The yellow module genes were profiled into five aspects, including the molecular function, cellular component, biological process, Kyoto Encyclopedia of Genes and Genomes (KEGG) pathway and WikiPathways (WP) pathway analysis. According to the KEGG pathway analysis, genes were mainly involved in the p53 signaling pathway and valine, leucine, and isoleucine degradation. The enrichment pathway ID was marked in the radar chart, where 1-P represented the radius of each enrichment. The radius was proportional to the enrichment significance (*P < 0.05; **P < 0.01; ***P < 0.001, Fig. 2E).

### Identification of the hub genes and in silico database validation

**Fig. 3:**
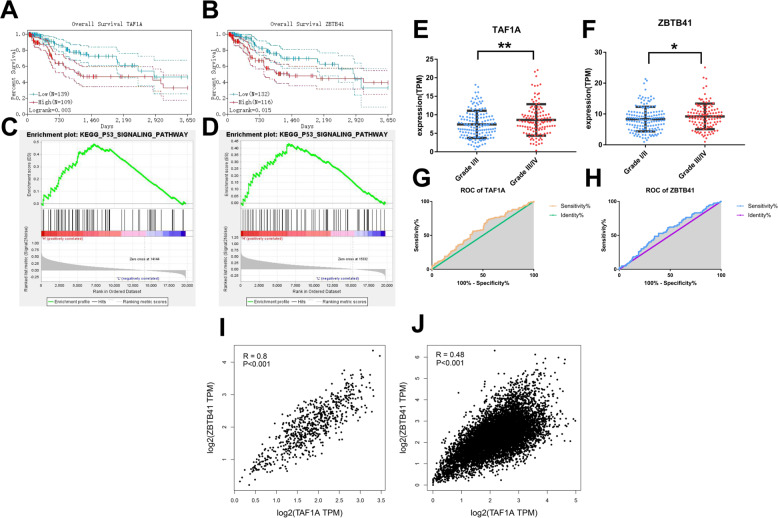
Overall survival (OS) of TAF1A and ZBTB41 in CC based on Kaplan–Meier plotter with a 95% confidence interval. The patients were stratified into the high-level and low-level groups according to the median expression. **A** TAF1A. **B** ZBTB41. Geneset enrichment analysis (GSEA) of CC in the TCGA database. FDR < 0.05 was set as the cut-off criteria. **C** “The p53 signaling pathway” enriched in samples with overexpressed TAF1A. **D** “The p53 signaling pathway” enriched in samples with overexpressed ZBTB41. The correlation of gene expression of TAF1A and ZBTB41 with pathological grades (I/II vs III/IV). The mRNA levels of TAF1A (**E**) and ZBTB41 (**F**). *P < 0.05, **P < 0.01. Receiver operator characteristic (ROC) curve analysis, high (III/IV) vs low (I/II) pathological grades. **G** TAF1A, **H** ZBTB41. Correlation analyses between TAF1A and ZBTB41 in CC (**I**) and the other 32 types of cancer (**J**) from the TCGA database.

The study samples were divided into various groups according to the expression of each candidate gene based on the expression data and clinical information of CC in the TCGA database. Survival analyses were carried out using the Kaplan–Meier method at a 95% confidence interval, and the significances were determined by the log-rank test. The Receiver operating characteristic (ROC) curve analyses were performed to determine the diagnostic efficiency of the high and low pathological grades of cancer. Two genes in the yellow module (TAF1A and ZBTB41) were negatively associated with patients’ overall survival (OS) based on multiple comprehensive analysis data (Fig. 3A, B).

The Geneset enrichment analysis (GSEA) was performed to obtain further validation of the functional enrichment of TAF1A and ZBTB41. Based on the cut-off criteria of false discovery rate (FDR) < 0.05, we found that “the p53 signaling pathway” was enriched significantly in both TAF1A and ZBTB41 highly expressed samples, compared to the low-expression samples (Fig. 3C, D). These results were consistent with previous enrichment results (Fig. 2E). Therefore, we focused on the p53 signaling pathway as the follow-up research content.

Furthermore, the TAF1A and ZBTB41 were significantly overexpressed in 304 high-grade tumour samples retrieved from the TCGA database (*P < 0.05, **P < 0.01, Fig. 3E, F). Besides, ROC curve analysis showed a modest but useful contribution of the TAF1A and ZBTB41 genes to the diagnostic efficiency of the high and low pathological grades of cancer (grade III/IV vs. grade I/II, Fig. 3G, H).

Immunohistochemistry (IHC) data from the Human Protein Atlas (HPA) database demonstrated significantly higher protein levels in tumour tissues than in normal tissues (Supplementary Fig. 1). Additionally, the expression levels of the two genes demonstrated a strong correlation with CC (R = 0.8, *P* < 0.001) and the other 32 types of cancer (R = 0.48, *P* < 0.001, Fig. 3J).

### Experimental validation of TAF1A and ZBTB41

**Fig. 4:**
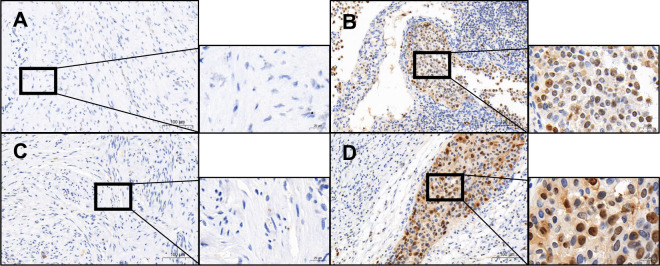
Immunohistochemistry results of clinical tissue specimens of patients. Representative photomicrographs of TAF1A stained tumours (**B**) and adjacent tissues (**A**). Representative photomicrographs of ZBTB41 stained tumours (**D**) and adjacent tissues (**C**).

**Fig. 5:**
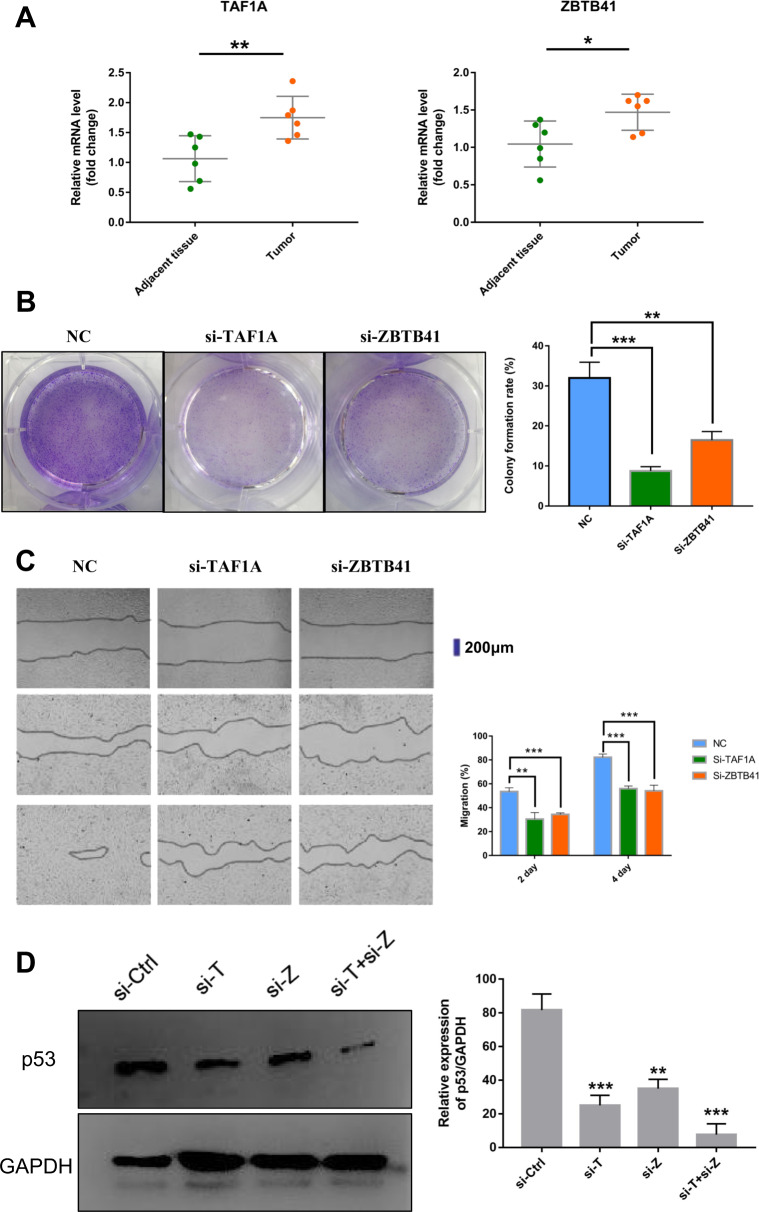
In vitro study of TAF1A and ZBTB41. **A** Relative mRNA expression of TAF1A and ZBTB41 in CC and adjacent tissues. **B** Clone formation assays of siControl, siTAF1A and siZBTB41 in the HeLa cell line. **C** Scratch wound healing assay of siTAF1A and siZBTB41 in the HeLa cell line. **D** Western blot was used to detect the expression of p53 protein in Hela cells after the treatment of siTAF1A, siZBTB41 and siTAF1A+siZBTB41, and the relative expression of p53 protein compared with GAPDH. Student’s *t*-tests were used to evaluate the statistical significance of differences. *P < 0.05; **P < 0.01, ***P < 0.001.

The IHC staining results showed that TAF1A and ZBTB41 were significantly overexpressed in the CC tissues than in the corresponding normal tissues (Fig. 4A–D and Supplementary Fig. 2, *P* < 0.001). More convincingly, the qRT-PCR clinical tissue samples showed a significant upregulation of TAF1A (*P* < 0.01) and ZBTB41 (*P* < 0.05) in tumours compared to the normal healthy tissues (Fig. 5A).

Furthermore, clone formation assays in the HeLa cell line (cervical cancer cell line) revealed that TAF1A and ZBTB41 knockdown significantly inhibits cell proliferation (Fig. 5B). Additionally, the wound scratch healing assay showed that TAF1A and ZBTB41 knockdown inhibits tumour cell migration (Fig. 5C). On the other hand, the p53 protein expression was significantly reduced following TAF1A or ZBTB41 knockdown and the simultaneous knockdown of both genes in the HeLa cell line (Fig. 5D). This result verifies our previous prediction (Fig. 2E and Fig. 3C, D).

As TAF1A and ZBTB41 were significantly co-expressed, we sought to determine whether TAF1A interacts with ZBTB41, and to explore the influence of the activity of the p53 signaling pathway on their interaction ability. We used the PFTα as an inhibitor of the p53 signaling pathway based on the published literature [14, 15].

**Fig. 6:**
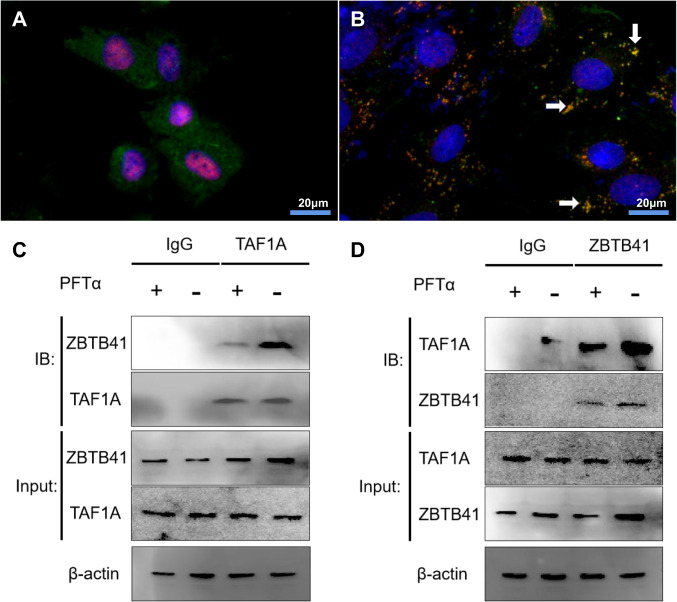
The IF of HeLa cell and Co-IP of TAF1A and ZBTB41. **A** Microscopic imaging of cells after 24 h of PFTα treatment. **B** HeLa cell microscopic imaging with DMSO treatment. Red: TAF1A; Green: ZBTB41; Blue: DAPI; magnification ×400. **C**, **D** Co-IP with endogenous proteins indicated that ZBTB41 interacted with TAF1A. DMSO was the vehicle (–) or PFTα (+, 10 μM) for 24 h. Cell lysates were immunoprecipitated with normal IgG, TAF1A (**C**), or ZBTB41 (**D**) antibody. The immunoprecipitates were blotted (IB) with ZBTB41 (**C**) or TAF1A (**D**) antibody. The PFTα suppressed the interaction between ZBTB41 and TAF1A.

Immunofluorescence results showed no significant intracellular overlap between TAF1A (red fluorescence) and ZBTB41 (green fluorescence) in the PFTα-treated experimental group (Fig. 6A). However, a significant intracellular overlap between the TAF1A and ZBTB41 was observed in the control group (without PFTα) (Fig. 6B, indicated by the arrow). This confirms that the PFTα inhibits the spatial cross-linking of the TAF1A and ZBTB41.

A Co-immunoprecipitation (Co-IP) assay using endogenous proteins extracted from the vehicle- or PFTα-treated HeLa cells showed that the ZBTB41 and TAF1A were coimmunoprecipitated(Fig. 6C, D), demonstrating they form a complex in Hela cells (Supplementary Fig. 3, *P* < 0.01). Their interaction was significantly suppressed by PFTα, which was likely attributable to the malignancy of cancer cells influenced by p53 signaling pathway.

### Ab initio protein prediction and model test

**Fig. 7:**
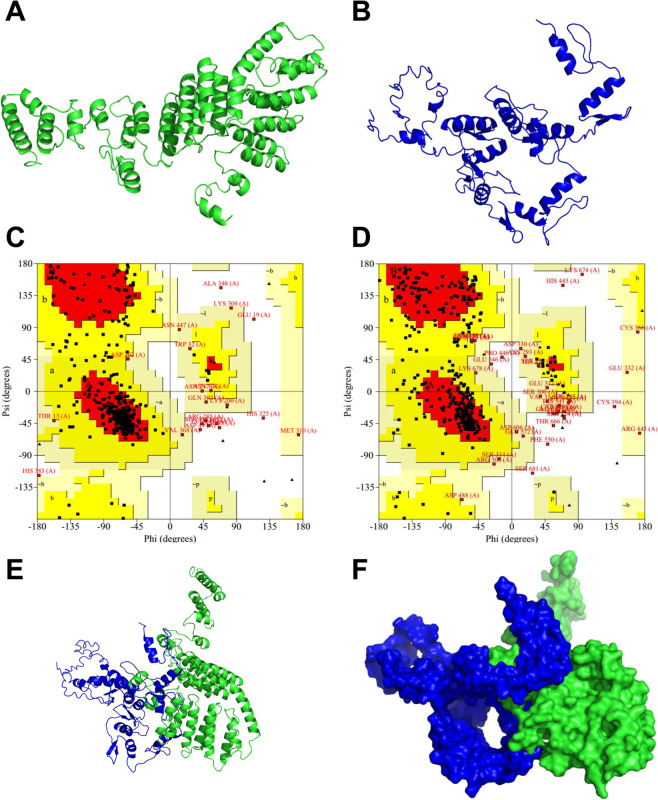
Model construction and molecular docking of TAF1A and ZBTB41. **A** The 3D protein structure of TAF1A (green). **B** The 3D protein structure of ZBTB41 (blue). **C** A Ram achandran plot of TAF1A protein. **D** A Ram achandran plot of ZBTB41 protein. Red is the best area; Yellow is the general area; White is not an allowed area. Ribbon structure (**E**) and 3D surface structure (**F**) model schematic diagrams of docking between TAF1A (green) and ZBTB41 (blue).

The I-TASSER server modeling showed TAF1A protein as a globular protein, and its secondary structure mainly consisted of a spiral and random curl (Fig. 7A). After energy minimisation and optimisation, the Ramachandran plot’s allowable range was 97.6% (above the limit of 95%), which could be used for subsequent molecular docking (Fig. 7C). The core structure of the ZBTB41 protein was composed of the BTB domain. The modeling results showed that it was a globular protein, and its main secondary structure was composed of long helix, folding and random curl (Fig. 7B). The Ramachandran plot’s allowable range was 96.9% (above the limit of 95%), indicating its usability in subsequent molecular docking studies (Fig. 7D).

### Molecular docking of TAF1A with ZBTB41

**Fig. 8:**
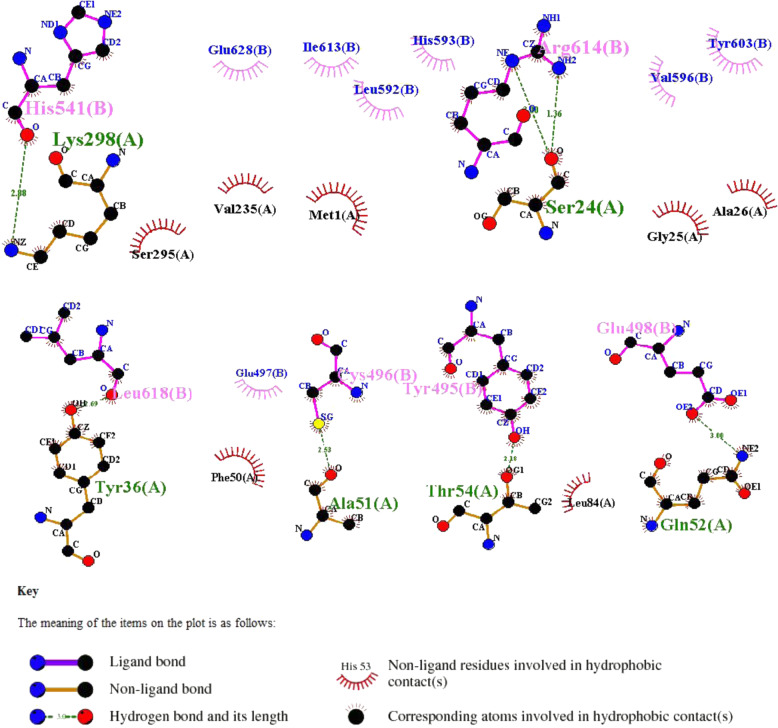
The interaction site and force between TAF1A and ZBTB41. The 2D representation of Ligplot analysis: TAF1A (A)–ZBTB41 (B). Tyr36 (A) and Leu618 (B) formed a hydrogen bond; Ala51 (A) and Cys496 (B) formed a hydrogen bond; Thr54 (A) and Tyr495 (B) formed a hydrogen bond; Gln52 (A) and Glu498 (B) formed a hydrogen bond; Lys298 (A) and His541 (B) formed a hydrogen bond; Ser24 (A) and Arg614 (B) formed two hydrogen bonds.

To further explore their structure-activity relationship, docking analysis demonstrated that TAF1A docked with high affinity to the BTB domain of ZBTB41 (Fig. 7E, F). We selected the lowest energy conformation and then simulated a three-dimensional structure of TAF1A-ZBTB41 protein complex. Moreover, the TAF1A (A) and ZBTB41 (B) interacted via hydrophobic, Van der Waals, hydrogen bonding and electrostatic. Furthermore, seven hydrogen bonds formed between Lys298 (A) and His541 (B), Ser24 (A) and Arg614 (B), Tyr36 (A) and Leu618 (B), Ala51 (A) and Cys496, Thr54 (A) and Tyr495 (B), and Gln52 (A) and Glu498 (B). The formation of these hydrogen bonds increased the ability of the two proteins to target each other, thereby acting act as a signal for protein activation (Fig. 8).

## Discussion

Cervical cancer (CC) is a heterogeneous disease accompanied by a high clinical recurrence and metastasis rate [16]. The prognostic evaluation indices of CC mainly include the FIGO stage, lymph node metastasis and interstitial infiltration depth, among others [17]. However, these features are not accurate enough for prognostic evaluation. Therefore, it is imperative to find biomarkers that can accurately guide the treatment and management of CC [18]. In the current study, we have found unique molecules associated with the pathological grade of CC, which have been experimentally validated to a certain extent.

Similar studies have shown altered gene expression profiles in gynecological tumors, including CC compared with the normal tissues [19, 20]. Among the differentially expressed genes, the hub genes are correlated with the pathogenesis and progression of CC. The WGCNA analysis can systematically demonstrate the interaction patterns among genes and improve large-scale data analysis efficiency better than the traditional method. We adopted the strategy of integrated data analysis combined with experimental verification to increase confidence in the results.

Previously, WGCNA has been widely used in microarray data analysis for gene annotation, prediction of potential gene function, discovery of new biomarkers for diseases, identification and validation of chemotherapy drugs [21–23]. We comprehensively analysed the expression data of large-scale genes and selected two candidate genes (TAF1A and ZBTB41) with significantly correlated expression profiles.

The TATA-Box Binding Protein Associated Factor, RNA Polymerase I Subunit A (TAF1A) is a protein-coding gene component of the SL1/TIF-IB transcription factor complex playing involved in the assembly of the RNA polymerase I preinitiation complex. It can recruit RNA polymerase I to ribosomal DNA promoters, which is the essential for ribosomal biogenesis [24]. A previous study has shown that TAF1A can interact with RRN3, and play a synergistic role in the disruption of the nucleolar structure caused by the segregation of the nucleolar components [25]. In dilated cardiomyopathy, compound heterozygous recessive mutations in TAF1A exacerbate fibrosis of the explanted hearts. Additionally, in zebrafish, a knockout of the homologous gene recapitulates a heart failure phenotype, pericardial oedema, decreased ventricular systolic function, and embryonic mortality [26].

On the other hand, Zinc Finger and BTB Domain Containing 41 (ZBTB41) is an important paralog of the Zinc Finger Protein 407 (ZNF407). The BTB domain of the ZBTB41 is highly conserved and mediates protein–protein interactions. The BTB is currently known to play an important role in transcriptional regulation, chromatin remodeling, protein degradation, and cytoskeletal regulation. It is also closely associated with functions of mammalian growth and development, including lymphocyte development, axonal orientation, gonadal morphogenesis, among others [27]. The proliferation and migration of cancer cells, such as those of the human acute promyelocytic leukemia, lymphoma, and prostate cancer, are also closely related to the BTB protein [28–30]. In hepatocellular carcinoma, ZBTB41 has been found to be a hub gene that was upregulated and regulated of the expressions of several key miRNAs [31].

p53, a transcriptional factor, has been generally viewed as an effective anticancer target. It is mutated in over 50% of the human tumours and is related to rapid tumour progression and resistance to anticancer therapy [32]. Pifithrin-α (PFTα) is a stable water-soluble compound with a molecular weight of 367, which has been used extensively as an inhibitor of the p53 signalling pathway [33–35]. The structural formula of the PFTα is shown in Supplementary Fig. 4A. The PFTα can blocks the p53-dependent transcriptional activity and reduces the p53 response genes’ activity in endogenous cells, including cyclin G, p21, and mdm2 [36]. Besides, PFTα regulates the nuclear import or/and export of the p53 protein [37].

Moreover, the PFTα inhibits the heat shock proteins, glucocorticoid receptor signal transduction, and the NF-κB activity, and blocks the induction of some receptors, such as the androgen receptor and CD95 [38–40]. These mechanisms indicate that the PFTα activity is not limited to the p53-related pathways but involves other cytokines and signal transduction pathways. Therefore, the PFTα can be used in the laboratory to characterise p53-mediated events, which can ameliorate chemotherapy- and radiotherapy-associated side effects in patients [41].

Western blot analyses demonstrated a dose-dependent inhibitory effect of the PFTα on the p53 protein HeLa cells (Supplementary Fig. 4B). This finding justified the usage of PFTα for our subsequent experiments. In this study, cytological experiments revealed that the TAF1A and ZBTB41 might be involved in the proliferation and migration of tumour cells, and that they may interact endogenously. The interaction between TAF1A and ZBTB41 can be weakened by the PFTα, which inhibits the p53 signaling pathway. Therefore, we believe that TAF1A and ZBTB41 proteins function by interacting with each other in vivo and may be related to the regulatory mechanism of the p53 signaling. The specific molecular regulatory mechanisms may be a key marker for the pathogenesis of CC.

Molecular modeling and docking techniques have been successfully used to study protein–ligand interactions and protein function [42, 43]. Since the structures of TAF1A and ZBTB41 proteins have not been determined, a more widely applicable ab initio modeling technique was used in this study. Protein interaction sites are generally located in a bioactive region of the protein surface. Such molecular details may be the underlying mechanism by which the two proteins target each other, thereby activating the corresponding protein signaling pathways. The TAF1A-ZBTB41 protein complex model would provides a framework for further studies on their molecular dynamics.

Further studies aimed at inducing site-directed mutations at the sites where TAF1A and ZBTB41 interact or designing targeted molecules to block their interaction in a mouse model are encouraged. This can precisely interfere with the molecular basis of tumour development while reducing toxic side effects. Our study provides an important reference for future analysis of tumour occurrence mechanisms.

Based on the obtained results, we speculate that TAF1A and ZBTB41 may be important biomarkers of CC, and their functions may be interdependent. We present new insights that these identified molecules could potentially serve as keys to understanding the underlying mechanism of CC pathogenesis.

## Materials and methods

### Data procession

The gene expression profiles of CC were extracted from the Metabolic gEne RApid Visualizer (MERAV, http://merav.wi.mit.edu) database. This dataset included 28 CC cases of different stages and pathological grades. After all data filtering and background noise correction, the “affy” package from Bioconductor (http://www.bioconductor.org) was used to normalize and summarize. The top 25% most variant genes were then selected for subsequent analysis.

### Establishment of the weighted co-expression network analysis (WGCNA)

The R package “WGCNA” was used to construct the gene co-expression network analysis. To ensure a scale-free network distribution, WGCNA needs to select the appropriate weighted coefficient “β” using the “pickSoftThreshold” function. The correlation coefficient and the means of gene connectivity were calculated from 1 to 20, respectively. The selection criteria of β should give a correlation coefficient square that is greater than 0.9 and should guarantee a certain degree of genes connectivity. In the presented study, the soft-thresholding parameter β = 8 was selected. The dynamic cutting method was used to identify the co-expression module. The automatic network building function “blockwiseModules” was used to build the network according to the topological overlap measure (TOM) based on dissimilarity measure [44]. Minimum module size was 30, and the mergeCutHeight was 0.25 to merge modules with 0.75 similarity. All the other parameters were used in their default state.

### Module identification and gene function analysis

In this study, the module with the highest absolute correlation coefficient and *p* < 0.05 was selected as the significant module to determine highly correlated genes with CC phenotypes. The selected gene module was then annotated, integrated, and analysed using g:Profile (https://biit.cs.ut.ee/gprofiler/gconvert.cgi) at default settings. The enrichment comprised five categories: molecular function, cellular component, biological process, KEGG pathway and WP. The results were categorised according to the cut-off criterion of the adjusted *P* < 0.05 and demonstrated in a radar chart. A value of 1-P represented the radii of each group. The full names corresponding to the pathway ID are presented in the supplementary Table 2.

Considering the interaction of genes within the modules, we used the co-expression analysis method and a circos plot to illustrate the genes’ interaction within the three most significant modules.

### Hub gene identification and validation

The validation RNASeq data and the corresponding clinical information were extracted from the Cancer Genome Atlas Project (TCGA, https://cancergenome.nih.gov/) database and normalized using the edgeR package. Each candidate gene identified in the selected module was assessed using survival analysis and receiver operating characteristic (ROC) analysis. Samples with missing information were excluded. Correlation analyses were performed across the CC samples and the other 32 types of cancer from TCGA database (Supplementary Table 3 and 4). After verification, we selected the genes that had a significant impact for further analysis.

The CC samples in the TCGA dataset were divided into high-expression and low-expression groups according to the median expression values of each selected gene. To verify the pathway enrichment of the candidate genes in the TCGA data set, Geneset enrichment analysis (GSEA) was performed using data from the KEGG database, and compared with the previous results. FDR < 0.05 was set as the cut-off criteria. Subsequently, we measured the expressions of hub genes by the immunohistochemistry using the Human Protein Atlas (HPA, http://www.proteinatlas. org). The GSEA was performed using GSEA software (http://www.broadinstitute.org/gsea). Other analyses were conducted using custom-written code in R software.

### Preparation of the clinical samples of CC

Twenty CC patients hospitalised in the affiliated Zhuzhou hospital Xiangya medical college from 1 January 2019 to 1 January 2020 were randomly selected to verify the candidate biomarkers. All patients had complete hospital information and surgical records. Each histology diagnosis was confirmed by two pathologists independently, and the summary of patients’ information has been listed (Supplementary Table 5). The well-preserved CC and corresponding adjacent tissues were stored according to the standard experimental procedures. The Ethics committee of Zhuzhou central hospital, south-central university (Zhuzhou, China) approved the research protocol. Informed consent was obtained from all subjects.

### Immunohistochemistry (IHC)

We measured the expression of hub genes in clinical specimens by the immunohistochemistry. The paraffin-embedded CC tissue and the corresponding paracancerous tissue samples were deparaffinised in xylene and hydrated in graded ethanol concentrations in descending order. The endogenous peroxidase was removed with 3% H_2_O_2_ after antigen repair with sodium citrate solution. The sections were then treated with 3% of BSA to reduce non-specific staining. Subsequently, the sections were incubated with a primary antibody (1:200 dilutions) at 4 °C overnight, and then incubated with a secondary antibody at 37 °C for 40 min. The target proteins were visualized using 3,3’-diaminobenzidine as the colour substrate.

### RNA extraction and qRT-PCR

Quantitative real-time PCR (qRT-PCR) experiments were performed with the SYBR green-based detection system. Total RNA was extracted using an RNA extraction kit (TaKaRa Bio, China). cDNA synthesis was carried out with PrimeScript RT Master Mix (TaKaRa Bio, China), and the amplified products were detected by using SYBR pre-mix EX Taq (TaKaRa Bio, China). The qRT-PCR was performed on a 7500 real-time PCR system (Applied Biosystems, USA) with the primer shown in Supplementary Table 6.

### Cell culture and small interfering RNA (siRNA) transfection

The human cervical carcinoma cell line (HeLa) was purchased from the ATCC (American Type Culture Collection, Manassas, USA). The siTAF1A and siZBTB41 synthesized by RiboBio Co. (Guangzhou, China) were selected for transient knockdown experiments, which were determined by qRT-PCR and western blot (Supplementary Fig. 5). The siControl was used as a control, and siRNA transfections were performed using Lipofectamine 2000 (Thermo Fisher Scientific), according to the manufacturer’s instructions.

### Proliferation and migration analysis

Cell proliferation was measured by colony formation experiment. Different groups of cells were inoculated into a six-well plate at a density of (5 × 10^3^ cells per well). After 4 days, the cells were fixed with 4% paraformaldehyde for 15 min, and then stained with 1% crystal violet. A wound scratch healing assay was used for determining the migration of cells. Briefly, the cultured cells were allowed to reach confluence on six-well culture plates, and a wound was created by scraping the middle of the cell monolayer across the entire diameter of the well with a P10 pipette tip. Floating cells were washed twice with Phosphate-Buffered Saline (PBS). Experiments were carried out without any proliferation inhibitor. Photographic images were captured using an inverted microscope to monitor the number of colonies (>50 cells) and the wound closure, and then analysed with ImageJ software (version 1.46q, http://rsbweb.nih.gov/ij/). All assays were conducted more than two times.

### Immunofluorescence (IF) localization

The PFTα, an inhibitor of the p53 signaling pathway, was purchased from Sigma–Aldrich (USA). Briefly, 2 × 10^6^ cells were seeded on coverslips in each well of a six-well plate. In another set of experiments, cells were incubated with 10 μM of PFTα for 24 h. The cells were washed thrice with PBS and fixed with 4% paraformaldehyde. The cells were blocked with PBS containing 1% of the goat serum for 30 min and incubated with a primary antibody overnight at 4 °C. The cells were washed six times with PBS and incubated with a secondary antibody for 1 h at room temperature. The stained cells were visualized using an Olympus BX61 microscope (Olympus Corporation, Tokyo, Japan).

### Reagent processing and Co-immunoprecipitation (Co-IP)

HeLa cells were seeded in 10 cm^2^ cell culture dishes (2 × 10^6^ cells) for 24 h, and then treated with PFTα 10 μM for 24 h. Dimethylsulfoxide (DMSO) was used as the control reagent. Subsequently, the medium was removed, and cells were washed thrice with PBS.

The RIPA lysate containing protein inhibitors was added, and the dishes were then placed on ice to lyse cells for 60 minutes. The lysates were collected into the EP tubes and centrifuged at 4 °C to remove the precipitation and retain the supernatant. The target antibody was added, and the setup was incubated at 4 °C for 2 h. After that, protein A/G-beads were added and incubated overnight at 4 °C. After the immunoprecipitation reaction, the tubes were centrifuged to precipitate the beads bound to the Ag–Ab complex. The collected protein A/G-beads were cleaned, SDS buffer was added, and boiled for 5–10 min. Western blotting was then performed to detect the proteins. Simultaneously, the study sample and IgG antibody served as the positive and negative controls, respectively. The primary and secondary antibodies against TAF1A, ZBTB41, and IgG were obtained from Invitrogen (Carlsbad, CA).

### Western blotting

Protein concentration was measured using BCA Protein Assay reagent (Beyotime, Shanghai, China). Samples were separated on an SDS-polyacrylamide gel and blotted to poly-vinylidene difluoride (PVDF) membranes. Signal detection was performed with ECL western blotting detection reagent (Thermo, USA). Antibodies used were anti-TAF1A (Invitrogen, Carlsbad, CA), anti-ZBTB41 (Invitrogen, Carlsbad, CA), anti–GAPDH (Abcam, Cambridge, MA), β-actin (Abcam, Cambridge, MA), and anti–p53 (Abcam, Cambridge, MA).

### Prediction of the protein structure

For ab initio modeling, 3D structure predictions were carried out by the I-TASSER server (http://zhanglab.ccmb.med.umich.edu/I-TASSER/) online database [45]. The amino acid sequence of the candidate protein was obtained from the Universe Protein Resource (UniProt) (http://www.uniprot.org/). During the modeling process, the Procheck server (https://servicesn.mbi.ucla.edu/PROCHECK/) was used to established the optimal model with low energy based on relevant theories. After model construction, the Ramachandran plot of candidate protein structures was drawn to evaluate the quality of the prediction model.

### Molecular docking

The ZDOCK algorithm was used for protein complex simulation after predicting the protein structure [46]. All possible docking patterns in space were obtained by translating and rotating the two proteins, and each binding model was evaluated using an energy-based scoring function. We picked the lowest score basis model by docking 100 times.

### Statistical analysis

The Pearson’s correlation test and logistical regression analyses were performed to investigate the correlation between the candidate genes. The log-rank test was used to compare the survival curves. Two-tailed Student’s *t*-test was used, and *P* < 0.05 was considered statistically significant. Statistical analyses were performed on R software (version 3.5.0) and GraphPad Prism software version 7.0 (GraphPad Software, USA). Image analysis was performed using Image J (NIH). The schematic representation of this process is illustrated in Fig. 1A.

## Supplementary information

supplementary tables

Supplementary figure 1

Supplementary figure 2

Supplementary figure 3

Supplementary figure 4

Supplementary figure 5

supplementary figure legends

## Data Availability

The datasets used and/or analysed during the current study are available from the corresponding author on reasonable request.
