# Supplementary material for: TAF1A and ZBTB41 serve as novel key genes in cervical cancer identified by integrated approaches
Source: Cancer Gene Ther. 2020 Dec 12;28(12):1298–311. doi: 10.1038/s41417-020-00278-1 (PMC8636252; doi:10.1038/s41417-020-00278-1)
Supplement: Supplementary file 1 — supplementary tables [file 41417_2020_278_MOESM1_ESM.docx]

| **Subsequently table 1:Total genes of the yellow module** | | | | | | | | | | | |
| --- | --- | --- | --- | --- | --- | --- | --- | --- | --- | --- | --- |
| **Genes** | | | | | | | | | | | |
| VAMP4 | CDA | HHLA1 | RAPH1 | CRY1 | TTC33 | NFU1 | ARHGAP5 | ZCCHC10 | POGK | DHX29 | SNORA25 |
| B3GALNT1 | ZNF518A | PRMT3 | LINC01003 | CRYZ | PYY2 | PDCD4 | C8orf59 | PIGG | ZNF529 | IFT80 | SUMO1 |
| CTNNAL1 | ZBED4 | RIDA | CAPZA2 | LOC143188 | IFIT5 | GOLIM4 | PLPP6 | FNBP1L | ANKRA2 | SLC6A11 | NR4A3 |
| TNFSF9 | CRYZL1 | CDK8 | LINC01405 | FAM76B | YTHDF3 | STK39 | LOC403323 | AUH | HAMP | JADE3 | C2orf82 |
| FPGT | HS3ST2 | ADAM10 | MRS2 | C12orf66 | SHPRH | GLUD1 | DUOXA2 | TMEM38B | PTBP2 | BCAR3 | PLEKHA5 |
| TNFRSF10B | CASP8AP2 | ANAPC10 | RRN3P3 | SLC38A6 | ASF1A | GMFB | SMAD4 | MSL2 | BBS2 | HAUS1 | COG6 |
| SUCLG2 | ZNF627 | C1D | PHKG1 | LINC01341 | CHRDL2 | GNL1 | MAT2A | PRMT6 | ABRACL | TCF12 | TMEM135 |
| SUCLA2 | C1orf67 | PIBF1 | PAXIP1-AS2 | TEKT4 | NIPSNAP3A | AMY2B | MMP3 | DNAAF2 | CACNG8 | YWHAH | SLC25A32 |
| VNN1 | TIGD1 | SLC19A2 | TADA1 | TTC30B | TMEM251 | CD163L1 | RP9P | APPL2 | KLHL12 | ZNF84 | API5 |
| CCNG1 | LIPH | PAICS | BORCS7 | WDSUB1 | FBXO3 | CHAMP1 | ATF1 | RCBTB1 | RDX | MRPL50 | NEK4 |
| TAF1A | ARL13B | SPHAR | LRIG3 | PAQR3 | GBAS | LINC00176 | NDUFA5 | RAVER2 | RECQL | ARRDC3 | LOC729994 |
| CCNG2 | PRIMPOL | MTX2 | TRAPPC6B | EID2 | RPS6KC1 | TRIB2 | C2CD4B | PCMTD2 | SCOC | BMPR1A | FLJ23185 |
| ZNF468 | PAXIP1-AS1 | CETN3 | TTC8 | AP2B1 | SNORA71B | MRPS28 | ALDH7A1 | PRB1 | ABCE1 | UTP14C | C16orf87 |
| LOC90462 | HMGB3P30 | KIF1C | LEO1 | DDX3X | SNORA68 | NDUFAF4 | P4HA1 | PRCC | ACTB | FBXO30 | PMS1 |
| ZNF700 | ALCAM | ZNF274 | DNAAF1 | DMXL1 | RANBP6 | GZMM | HDGFRP3 | ZNF823 | SCT | MRFAP1L1 | INTS2 |
| ZFAND2A | ACSL3 | CLPX | ZFP3 | PSORS1C2 | ACAD8 | H1F0 | ASCC1 | RNPC3 | ZBED8 | GCFC2 | MRPL44 |
| CARMIL3 | MICU2 | IL24 | LRRC75A-AS1 | DLAT | DIEXF | HOXB2 | TMED7 | CDKN2AIP | TRAPPC2 | WRB | GTPBP10 |
| ANGEL2 | ZUFSP | ZNF644 | JSRP1 | DNASE1L3 | STAU2 | HOXB3 | RLIM | KIF21A | MOAP1 | LOC391142 | RNF14 |
| TCEAL8 | RRAS2 | FAR1 | LYPLAL1 | DNMT3B | IL17B | HOXC10 | KCTD3 | KRBOX4 | C17orf75 | DHX29 | TTI1 |
| CD1A | R3HDM2 | HSDL2 | NUP35 | AGL | ZNF140 | PRMT1 | INSIG2 | RCOR3 | TFB2M | IFT80 | PREPL |
| AIFM1 | WDR47 | EFCAB2 | MITD1 | EEF1A1 | VEZF1 | HSF2 | DBR1 | ZNF83 | ATL2 | SLC6A11 | YEATS4 |
| SLC33A1 | LIMCH1 | LLPH | SETD9 | TIA1 | ZNF195 | HSPA9 | TUBE1 | CMTR2 | RFWD2 | CEP57 | PPIL4 |
| GORAB | FAM179B | PCGF5 | NUDCD2 | C1QBP | ZNF217 | HSPE1 | CRNKL1 | FGD6 | NFKBIZ | STARD3NL | VPS45 |
| NAF1 | PHF8 | EFCAB7 | HINT3 | TPSAB1 | SLC30A1 | S100A7A | COMMD10 | KDM3A | ZFP62 | CHUK | SSB |
| PXYLP1 | FRMD4B | GON7 | VPS37A | TSC2 | FASTKD3 | ENPP7 | SUCO | ZNF302 | ACTR6 | MAP3K7 |  |
| MOB1B | PPIP5K2 | ZNF559 | HIBADH | LOC728678 | KREMEN2 | ZNF260 | FAM8A1 | CTPS2 | SGCB | KDM6A |  |
| ZNF561 | CAMSAP2 | CAT | FGFR1OP | TAF1L | CDC73 | ID2 | OTUD6B | AZU1 | RBMY3AP | PSCA |  |
| ARHGAP12 | ZCCHC11 | MAP4K3 | NUDT3 | FUNDC1 | RNF219 | ACADM | MRPS23 | FEM1C | ISL2 | SMIM20 |  |
| GGPS1 | CLASP1 | PPM1D | ANXA10 | SOAT1 | VCPKMT | ZBTB41 | ERAP1 | MFF | USP46 | TRMT13 |  |
| TBPL1 | ABCB10 | TMEM128 | CHML | BNIP3 | ARMT1 | TEX9 | KDM3B | PYY | RFX7 | NCEH1 |  |
| MINPP1 | PDHX | REPS1 | CFAP36 | SPAST | HSPBAP1 | KCNS1 | PEX13 | PELI2 | REEP1 | MARCKSL1 |  |
| PREPL | YEATS4 | PPIL4 | VPS45 | SSB | PSCA | C2CD4B | PHKG1 | MRS2 | RAPH1 | CAPZA2 |  |

**Subsequently table 2: Full names of the corresponding pathway ID**

KEGG: 00280: Valine, leucine and isoleucine degradation

KEGG: 04115: p53 signaling pathway

WP: WP4536: Genes related to primary cilium development (based on CRISPR)

WP: WP4236: Disorders of the Krebs cycle

WP: WP3925: Amino Acid metabolism

GO: 0008757: S-adenosylmethionine-dependent methyltransferase activity

GO: 0008171: O-methyltransferase activity

GO: 0016273: arginine N-methyltransferase activity

GO: 0043933: protein-containing complex subunit organization

GO: 1903608: protein localization to cytoplasmic stress granule

GO: 0044424: intracellular part

GO: 0043229: intracellular organelle

GO: 0043231: intracellular membrane-bounded organelle

| **Subsequently table 3: Selected cancer types from TCGA** | | | | |
| --- | --- | --- | --- | --- |
| **TCGA Tumor** | | | | |
| ACC | BLCA | BRCA | CESC | CHOL |
| COAD | DLBC | ESCA | GBM | HNSC |
| KICH | KIRC | KIRP | LAML | LGG |
| LIHC | LUAD | LUSC | MESO | OV |
| PAAD | PCPG | PRAD | READ | SARC |
| SKCM | STAD | TGCT | THCA | THYM |
| UCEC | UCS | UVM |  |  |

| **Subsequently table 4: Abbreviations** | |
| --- | --- |
| ACC | Adrenocortical carcinoma |
| BLCA | Bladder Urothelial Carcinoma |
| BRCA | Breast invasive carcinoma |
| CESC | Cervical squamous cell carcinoma and endocervical adenocarcinoma |
| CHOL | Cholangiocarcinoma |
| COAD | Colon adenocarcinoma |
| DLBC | Lymphoid Neoplasm Diffuse Large B-cell Lymphoma |
| ESCA | Esophageal carcinoma |
| GBM | Glioblastoma multiforme |
| HNSC | Head and Neck squamous cell carcinoma |
| KICH | Kidney Chromophobe |
| KIRC | Kidney renal clear cell carcinoma |
| KIRP | Kidney renal papillary cell carcinoma |
| LAML | Acute Myeloid Leukemia |
| LGG | Brain Lower Grade Glioma |
| LIHC | Liver hepatocellular carcinoma |
| LUAD | Lung adenocarcinoma |
| LUSC | Lung squamous cell carcinoma |
| MESO | Mesothelioma |
| OV | Ovarian serous cystadenocarcinoma |
| PAAD | Pancreatic adenocarcinoma |
| PCPG | Pheochromocytoma and Paraganglioma |
| PRAD | Prostate adenocarcinoma |
| READ | Rectum adenocarcinoma |
| SARC | Sarcoma |
| SKCM | Skin Cutaneous Melanoma |
| STAD | Stomach adenocarcinoma |
| TGCT | Testicular Germ Cell Tumors |
| THCA | Thyroid carcinoma |
| THYM | Thymoma |
| UCEC | Uterine Corpus Endometrial Carcinoma |
| UCS | Uterine Carcinosarcoma |
| UVM | Uveal Melanoma |

| **Subsequently table 5: Summary of CC patient characteristics** | | | | |
| --- | --- | --- | --- | --- |
| **Patient** | **Age** | **Pathological pattern** | **Clinical stage** | **Pathological grade** |
| P01 | 45 | squamous carcinoma | IIa1 | III |
| P02 | 54 | adenocarcinoma | Ib | II |
| P03 | 26 | Not Available | Ia1 | I |
| P04 | 53 | squamous carcinoma | Ib1 | III |
| P05 | 61 | squamous carcinoma | Ia1 | I |
| P06 | 51 | squamous carcinoma | IIa2 | III |
| P07 | 60 | squamous carcinoma | Ia2 | II |
| P08 | 63 | squamous carcinoma | IIa1 | II |
| P09 | 34 | squamous carcinoma | Ia1 | I |
| P10 | 45 | squamous carcinoma | Ia1 | I |
| P11 | 58 | squamous carcinoma | Ia1 | I |
| P12 | 79 | squamous carcinoma | IIIb | III |
| P13 | 61 | squamous carcinoma | IIb | III |
| P14 | 48 | squamous carcinoma | Ia1 | I |
| P15 | 62 | squamous carcinoma | Ib1 | III |
| P16 | 35 | squamous carcinoma | IIa2 | II |
| P17 | 63 | squamous carcinoma | Ia1 | II |
| P18 | 42 | squamous carcinoma | Ia1 | I |
| P19 | 66 | squamous carcinoma | IIa2 | II |
| P20 | 61 | squamous carcinoma | Ia1 | I |

| **Subsequently table 6: Primer pairs for RT-PCR. Base sequences for determining β-ACTIN, TAF1A and ZBTB41 mRNA expression** | | |
| --- | --- | --- |
| **Oligonucleotide** | **Upstream Sequence (5' -> 3')** | **Downstream Sequence (5' -> 3')** |
| β-ACTIN | GGGACCTGACTGACTACCTC | TCATACTCCTGCTTGCTGAT |
| TAF1A | GGAGCCCAAGAGGTACTCAC | CCCCAGTTTACGGTGTTCTTCT |
| ZBTB41 | TTGCTGTCGGCAGTAGTTATTT | GTGTTACGTGATCCAGGGTGA |
