# Supplementary figures and images for: TAF1A and ZBTB41 serve as novel key genes in cervical cancer identified by integrated approaches
Source: Cancer Gene Ther. 2020 Dec 12;28(12):1298–311. doi: 10.1038/s41417-020-00278-1 (PMC8636252; doi:10.1038/s41417-020-00278-1)

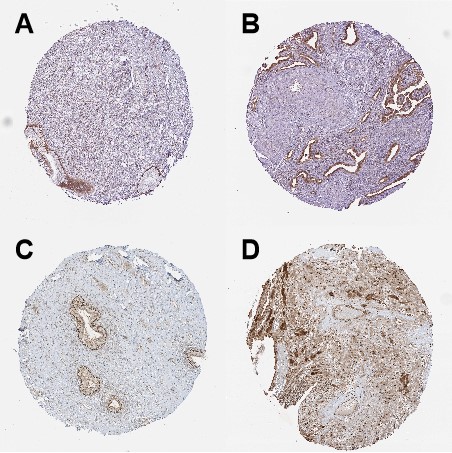

Supplement: Supplementary file 2 — Supplementary figure 1 [file 41417_2020_278_MOESM2_ESM.jpg]

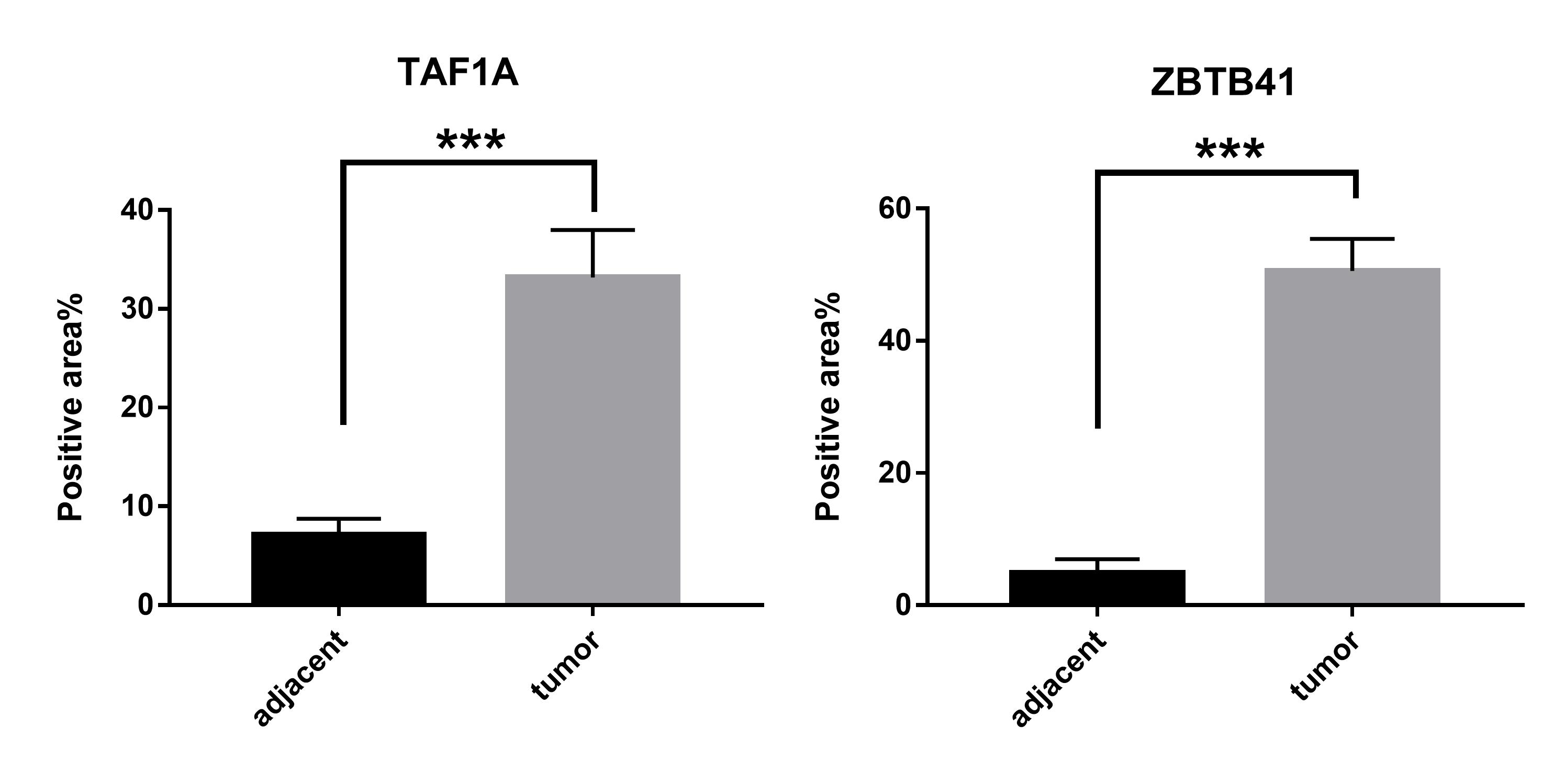

Supplement: Supplementary file 3 — Supplementary figure 2 [file 41417_2020_278_MOESM3_ESM.jpg]

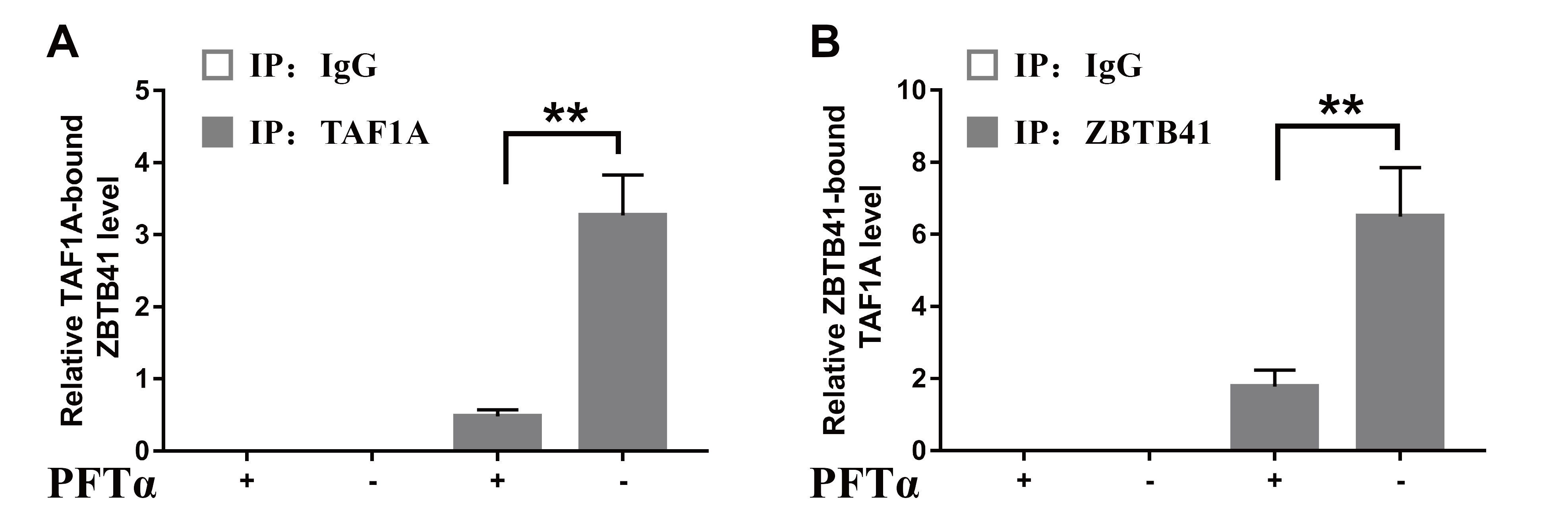

Supplement: Supplementary file 4 — Supplementary figure 3 [file 41417_2020_278_MOESM4_ESM.jpg]

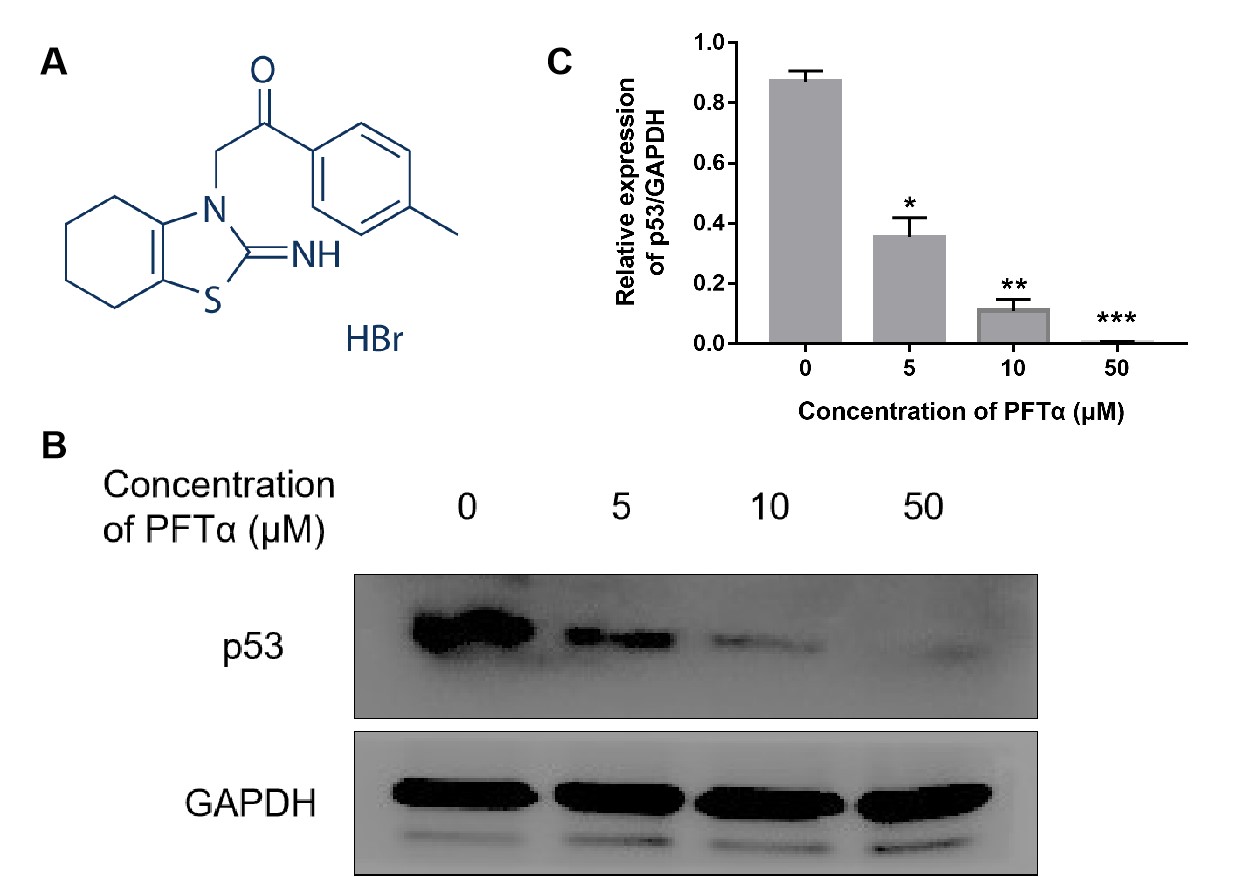

Supplement: Supplementary file 5 — Supplementary figure 4 [file 41417_2020_278_MOESM5_ESM.jpg]

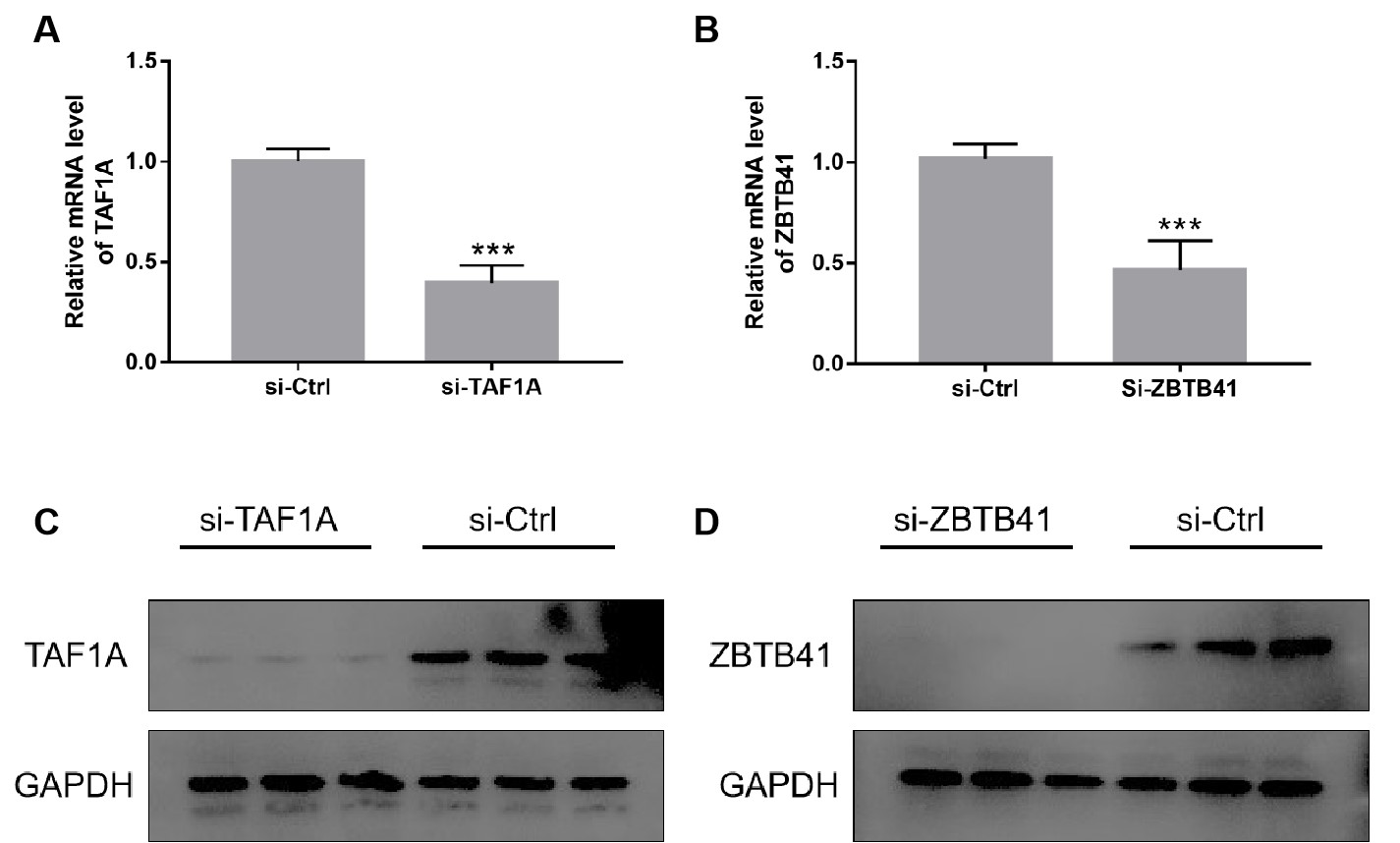

Supplement: Supplementary file 6 — Supplementary figure 5 [file 41417_2020_278_MOESM6_ESM.jpg]
