# Supplementary material for: TAF1A and ZBTB41 serve as novel key genes in cervical cancer identified by integrated approaches
Source: Cancer Gene Ther. 2020 Dec 12;28(12):1298–311. doi: 10.1038/s41417-020-00278-1 (PMC8636252; doi:10.1038/s41417-020-00278-1)
Supplement: Supplementary file 7 — supplementary figure legends [file 41417_2020_278_MOESM7_ESM.docx]

**SUPPLEMENTARY FIGURE 1 |** Immunohistochemistry of TAF1A and ZBTB41 based on the Human Protein Atlas.

1. Protein levels of TAF1A in the normal tissue (https://www.proteinatlas.org/ENSG00000143498-TAF1A/tissue/cervix%2C+uterine#img).
2. Protein levels of TAF1A in the tumour tissue (https://www.proteinatlas.org/ENSG00000143498-TAF1A/pathology/cervical+cancer#img).
3. Protein levels of ZBTB41 in the normal tissue (https://www.proteinatlas.org/ENSG00000177888-ZBTB41/tissue/cervix%2C+uterine#img).

(D) Protein levels of ZBTB41 in the tumour tissue (https://www.proteinatlas.org/ENSG00000177888-ZBTB41/pathology/cervical+cancer#img).

**SUPPLEMENTARY FIGURE 2 |**

Quantification of immunohistochemistry results: (A) TAF1A; (B) ZBTB41.

**SUPPLEMENTARY FIGURE 3 |**

Quantification of Co-IP results: Quantifications of ZBTB41 immunoprecipitated with TAF1A (A) and of TAF1A immunoprecipitated with ZBTB41 (B) as shown in figure 6 C and D, respectively. The proteins coimmunoprecipitated were normalized to β-actin. **p < 0.01 compared with TAF1A-immunoprecipitated (figure 6C) and ZBTB41-immunoprecipitated (figure 6D) groups with PFTα treatment (n = 3).

**SUPPLEMENTARY FIGURE 4 |**

1. The structural formula of PFTα.

(B) Western blot was used to detect the effect of different concentrations of PFTα on the expression of p53 protein in Hela cells. The inhibitory effect of PFTα on p53 was dose-dependent. 10μM PFTα can significantly inhibit the expression of p53. When the concentration was increased to 50μM, the expression of p53 diminished.

**SUPPLEMENTARY FIGURE 5 |**

1. qPCR was used to test the efficiency of si-TAF1A and si-ZBTB41 to knock down target genes.
2. Western blot detected the expression of the target protein after si-RNA knocked down the target gene. The results showed that si-RNA could effectively reduce the expression of mRNA and protein levels of target genes.
